# Supplementary material for: Swarm: robust and fast clustering method for amplicon-based studies
Source: PeerJ. 2014 Sep 25;2:e593. doi: 10.7717/peerj.593 (PMC4178461; doi:10.7717/peerj.593)
Supplement: Table S1 [file peerj-02-593-s002.pdf]

Domain	Genome Name	Genome Size (bp)	Phylum	Class
Bacteria	*Acidobacterium capsulatum* ATCC 51196	4,127,356	Acidobacteria	Acidobacteriae
	Akkermansia muciniphila ATCC BAA-835	2,664,102	Verrucomicrobia	Verrucomicrobiae
	Anaerocellum thermophilum Z-1320, DSM 6725	2,919,718	Firmicutes	Clostridia
	Bacteroides thetaiotaomicron VPI-5482	6,293,399	Bacteroidetes	Bacteroidia
	Bacteroides vulgatus ATCC 8482	5,163,189	Bacteroidetes	Bacteroidia
	Bordetella bronchiseptica RB50	5,339,179	Proteobacteria	Betaproteobacteria
	Burkholderia xenovorans LB400	973,113	Proteobacteria	Betaproteobacteria
	Caldicellulosiruptor saccharolyticus DSM 8903	2,970,275	Firmicutes	Clostridia
	Chlorobaculum tepidum TLS	2,154,946	Chlorobi	Chlorobia
	Chlorobium limicola DSM 245	2,763,181	Chlorobi	Chlorobia
	Chlorobium phaeobacteroides DSM 266	3,133,902	Chlorobi	Chlorobia
	Chlorobium phaeovibrioides DSM 265	1,966,858	Chlorobi	Chlorobia
	Chloroflexus aurantiacus J-10-fl	5,258,541	Chloroflexi	Chloroflexi
	Clostridium thermocellum ATCC 27405	3,843,301	Firmicutes	Clostridia
	Deinococcus radiodurans R1	3,284,156	Thermi	Deinococci
	Desulfovibrio desulfuricans ATCC 27774	2,873,437	Proteobacteria	Deltaproteobacteria
	Desulfovibrio piger ATCC 29098	2,826,240	Proteobacteria	Deltaproteobacteria
	Dictyoglomus turgidum DSM 6724	1,855,560	Dictyoglomi	Dictyoglomia
	Enterococcus faecalis V583	3,359,974	Firmicutes	Bacilli
	Fusobacterium nucleatum ATCC 25586	2,174,500	Fusobacteria	Fusobacteria
	Gemmatimonas aurantiaca T-27T	4,636,964	Gemmatimonadetes	Gemmatimonadetes
	Herpetosiphon aurantiacus ATCC 23779	6,785,430	Chloroflexi	Chloroflexi
	Hydrogenobaculum sp. Y04AAS1	1,559,514	Aquificae	Aquificae
	Leptothrix cholodnii SP-6	4,909,403	Proteobacteria	Betaproteobacteria
	Nitrosomonas europaea ATCC 19718	2,812,094	Proteobacteria	Betaproteobacteria
	Nostoc sp. PCC 7120	7,211,789	Cyanobacteria	unclassified
	Pelodictyon phaeoclathratiforme BU-1	3,018,238	Chlorobi	Chlorobia
	Persephonella marina EX-H1	2,467,104	Aquificae	Aquificae
	Porphyromonas gingivalis ATCC 33277	2,354,886	Bacteroidetes	Bacteroidia
	Rhodopirellula baltica SH 1	7,145,576	Planctomycetes	Planctomycetacia
	Rhodospirillum rubrum ATCC 11170	4,406,557	Proteobacteria	Alphaproteobacteria
	Ruegeria pomeroyi DSS-3	4,601,053	Proteobacteria	Alphaproteobacteria
	Salinispora arenicola CNS-205	5,786,361	Actinobacteria	Actinobacteria
	Salinispora tropica CNB-440	5,183,331	Actinobacteria	Actinobacteria
	Shewanella baltica OS185	5,312,910	Proteobacteria	Gammaproteobacteria
	Shewanella baltica OS223	5,358,884	Proteobacteria	Gammaproteobacteria
	Sulfitobacter sp. EE-36	3,547,243	Proteobacteria	Alphaproteobacteria
	Sulfitobacter sp. NAS-14.1	4,002,069	Proteobacteria	Alphaproteobacteria
	Sulfurihydrogenibium sp. YO3AOP1	1,838,442	Aquificae	Aquificae
	Sulfurihydrogenibium yellowstonense SS-5	1,534,471	Aquificae	Aquificae
	Thermoanaerobacter pseudethanolicus ATCC 33223	2,362,816	Firmicutes	Clostridia
	Thermotoga neapolitana DSM 4359	1,884,562	Thermotogae	Thermotogae
	Thermotoga petrophila RKU-1	1,824,357	Thermotogae	Thermotogae
	Thermotoga sp. RQ2	877,693	Thermotogae	Thermotogae
	Thermus thermophilus HB8	2,116,056	Thermi	Thermi
	Treponema denticola ATCC 35405	2,843,201	Spirochaetes	Spirochaetes
	Zymomonas mobilis ZM4	2,223,497	Proteobacteria	Alphaproteobacteria
Archaea	*Archaeoglobus fulgidus* DSM 4304	2,178,400	Euryarchaeota	Archaeoglobi
	Ignicoccus hospitalis KIN4/I	1,297,538	Crenarchaeota	Thermoprotei
	Methanocaldococcus jannaschii DSM 2661	1,664,970	Euryarchaeota	Methanococci
	Methanococcus maripaludis C5	1,780,761	Euryarchaeota	Methanococci
	Methanococcus maripaludis S2	1,661,137	Euryarchaeota	Methanococci
	Nanoarchaeum equitans Kin4-M	490,885	Nanoarchaeota	Nanoarchaea
	Pyrobaculum aerophilum IM2	2,222,430	Crenarchaeota	Thermoprotei
	Pyrobaculum calidifontis JCM 11548	2,009,313	Crenarchaeota	Thermoprotei
	Pyrococcus horikoshii OT3	1,738,505	Euryarchaeota	Thermococci
	Sulfolobus tokodaii 7(S311)	2,694,756	Crenarchaeota	Thermoprotei

Suppl. Tab. 1. Biological composition of the even and uneven mock-communities used for this study.
