# Supplementary material for: Swarm: robust and fast clustering method for amplicon-based studies
Source: PeerJ. 2014 Sep 25;2:e593. doi: 10.7717/peerj.593 (PMC4178461; doi:10.7717/peerj.593)
Supplement: Table S2 [file peerj-02-593-s003.pdf]

Divergence	Metric	Clustering algorithm				
		CD-HIT-454	DNAclust	Swarm	Usearch (no presorting)	Usearch (presorting)
1	*adjusted Rand index*	0.916	0.883	**0.958**	0.780	0.806
2		0.956	0.971	**0.972**	0.785	0.846
3		0.943	**0.979**	0.977	0.802	0.871
4		0.965	0.979	**0.981**	0.853	0.946
5		0.967	**0.984**	0.983	0.913	0.974
6		0.968	**0.985**	**0.985**	0.949	0.984
7		0.968	0.980	**0.985**	0.972	0.982
8		0.967	0.969	**0.986**	0.979	0.981
9		0.965	0.969	**0.983**	0.979	0.969
10		0.964	0.907	**0.983**	0.980	0.957
11		0.955	0.921	**0.983**	0.979	0.928
12		0.943	0.914	**0.981**	0.976	0.901
13		0.937	0.914	**0.981**	0.970	0.895
14		0.931	0.893	**0.978**	0.960	0.875
15		0.897	0.870	**0.977**	0.942	0.871
16		0.867	0.867	**0.974**	0.929	0.867
17		0.854	0.852	**0.978**	0.916	0.819
18		0.852	0.831	**0.976**	0.907	0.806
19		0.861	0.622	**0.958**	0.898	0.780
20		0.739	0.554	**0.973**	0.881	0.769
1	*precision*	0.993	0.993	0.992	**0.999**	0.996
2		0.992	0.992	0.992	**0.999**	0.992
3		0.992	0.992	0.992	**0.997**	0.993
4		**0.992**	**0.992**	**0.992**	**0.992**	**0.992**
5		0.992	0.992	0.992	**0.996**	0.991
6		0.991	0.991	**0.992**	0.991	0.991
7		0.991	0.986	**0.992**	0.991	0.986
8		0.987	0.972	**0.992**	0.990	0.983
9		0.980	0.971	**0.990**	**0.990**	0.970
10		0.979	0.932	**0.990**	**0.990**	0.963
11		0.978	0.932	**0.990**	0.985	0.940
12		0.964	0.931	**0.989**	0.981	0.927
13		0.954	0.930	**0.987**	0.976	0.918
14		0.948	0.914	**0.985**	0.965	0.907
15		0.932	0.901	**0.983**	0.958	0.899
16		0.918	0.898	**0.982**	0.942	0.893
17		0.899	0.880	**0.981**	0.931	0.875
18		0.897	0.871	**0.980**	0.924	0.863
19		0.887	0.726	**0.967**	0.909	0.841
20		0.803	0.644	**0.978**	0.897	0.833
1	*recall*	0.900	0.883	**0.949**	0.778	0.812
2		0.946	0.968	**0.970**	0.790	0.860
3		0.940	**0.981**	0.976	0.816	0.890
4		0.958	**0.983**	0.979	0.871	0.953
5		0.960	**0.986**	0.981	0.924	0.980
6		0.962	**0.988**	0.986	0.950	0.987
7		0.962	0.986	**0.987**	0.971	0.987
8		0.962	0.987	**0.988**	0.978	0.987
9		0.959	**0.988**	0.987	0.979	0.984
10		0.961	0.972	**0.987**	0.982	0.979
11		0.961	0.987	**0.988**	0.985	0.977
12		0.958	**0.988**	0.987	0.983	0.972
13		0.958	**0.988**	0.985	0.982	0.969
14		0.959	**0.986**	0.983	0.981	0.969
15		0.956	0.986	**0.987**	0.980	0.970
16		0.956	**0.988**	0.985	0.979	0.972
17		0.957	0.981	**0.988**	0.978	0.960
18		0.955	0.981	**0.987**	0.979	0.959
19		0.952	**0.981**	0.972	0.980	0.959
20		0.949	0.973	**0.985**	0.978	0.958

Suppl. Tab. 2. Uneven mock-community. Median values for Figure 2.
