# Supplementary material for: Swarm: robust and fast clustering method for amplicon-based studies
Source: PeerJ. 2014 Sep 25;2:e593. doi: 10.7717/peerj.593 (PMC4178461; doi:10.7717/peerj.593)
Supplement: Table S3 [file peerj-02-593-s004.pdf]

Divergence	Metric	Clustering algorithm				
		CD-HIT-454	DNAclust	Swarm	Usearch (no presorting)	Usearch (presorting)
1	*adjusted Rand index*	0.834	0.792	**0.891**	0.731	0.765
2		0.914	0.930	**0.942**	0.734	0.796
3		0.923	**0.948**	**0.948**	0.744	0.817
4		0.928	**0.961**	0.952	0.777	0.874
5		0.930	**0.964**	0.953	0.848	0.931
6		0.931	**0.965**	0.955	0.868	0.956
7		0.932	0.956	**0.961**	0.904	0.955
8		0.921	0.926	**0.958**	0.946	0.950
9		0.919	0.924	0.951	**0.952**	0.936
10		0.917	0.836	**0.960**	0.954	0.903
11		0.917	0.822	**0.952**	0.951	0.855
12		0.908	0.818	**0.950**	0.945	0.838
13		0.867	0.787	**0.950**	0.939	0.831
14		0.859	0.780	**0.954**	0.931	0.827
15		0.847	0.765	**0.942**	0.924	0.820
16		0.836	0.711	**0.935**	0.906	0.810
17		0.820	0.651	**0.925**	0.896	0.774
18		0.794	0.583	**0.941**	0.875	0.704
19		0.768	0.559	**0.925**	0.858	0.668
20		0.758	0.511	**0.925**	0.835	0.647
1	*precision*	0.992	0.994	0.989	**0.999**	0.998
2		0.990	0.989	0.989	**0.999**	0.997
3		0.989	0.989	0.989	**0.997**	0.995
4		0.989	0.989	0.989	**0.995**	0.992
5		0.988	0.988	0.988	**0.996**	0.988
6		0.987	0.988	0.988	**0.994**	0.987
7		**0.987**	0.974	**0.987**	**0.987**	0.975
8		0.970	0.932	**0.987**	**0.987**	0.969
9		0.961	0.929	0.985	**0.986**	0.950
10		0.959	0.887	0.985	**0.986**	0.914
11		0.959	0.850	0.984	**0.985**	0.891
12		0.946	0.837	**0.983**	0.982	0.870
13		0.923	0.819	**0.981**	0.971	0.861
14		0.916	0.809	**0.981**	0.962	0.857
15		0.904	0.791	**0.970**	0.948	0.846
16		0.888	0.769	**0.964**	0.929	0.842
17		0.864	0.736	**0.949**	0.915	0.809
18		0.852	0.667	**0.967**	0.897	0.768
19		0.828	0.651	**0.945**	0.875	0.729
20		0.816	0.630	**0.944**	0.858	0.714
1	*recall*	0.817	0.805	**0.900**	0.729	0.777
2		0.899	0.927	**0.944**	0.734	0.809
3		0.909	0.949	**0.951**	0.749	0.836
4		0.914	**0.967**	0.955	0.790	0.891
5		0.916	**0.970**	0.957	0.855	0.946
6		0.919	**0.972**	0.958	0.883	0.966
7		0.921	**0.971**	0.968	0.912	0.969
8		0.921	**0.969**	0.964	0.947	0.965
9		0.924	**0.970**	0.960	0.954	0.963
10		0.923	0.966	**0.969**	0.958	0.958
11		0.927	0.962	**0.963**	0.958	0.954
12		0.927	**0.966**	0.953	0.955	0.951
13		0.926	**0.968**	0.962	0.955	0.950
14		0.925	**0.969**	0.964	0.955	0.950
15		0.922	**0.966**	0.956	0.955	0.949
16		0.925	**0.963**	0.960	0.956	0.949
17		0.923	**0.963**	0.947	0.956	0.948
18		0.921	0.960	**0.961**	0.956	0.943
19		0.919	**0.967**	0.961	0.956	0.935
20		0.915	0.959	**0.960**	0.956	0.935

Suppl. Tab. 3. Even mock-community. Median values for Supplementary Figure 1.
