# Supplementary material for: Swarm: robust and fast clustering method for amplicon-based studies
Source: PeerJ. 2014 Sep 25;2:e593. doi: 10.7717/peerj.593 (PMC4178461; doi:10.7717/peerj.593)
Supplement: File S1 [file peerj-02-593-s008.zip › Supplementary_File_1.html]

xml version="1.0" encoding="utf-8"?


Swarm: robust and fast clustering method for amplicon-based studies


# Swarm: robust and fast clustering method for amplicon-based studies

Frédéric Mahé, Torbjørn Rognes, Christopher Quince, Colomban de Vargas
and Micah Dunthorn

Supplementary File 1

## Table of Contents

- 1 Disclaimer
- 2 Mock-communities analyses 
  - 2.1 Synopsis
  - 2.2 Download the datasets
  - 2.3 Dereplication
  - 2.4 Taxonomic assignment
  - 2.5 Clustering Quality Metrics
  - 2.6 Scripts 
    - 2.6.1 Shuffling
    - 2.6.2 Swarm breaker
    - 2.6.3 Confusion table
    - 2.6.4 Clustering metrics
  - 2.7 Visualization

## 1 Disclaimer

The purpose of this document is too provide the reader with details on
the bioinformatics methods we used to prepare the paper "Swarm: robust
and fast clustering method for amplicon-based studies". The code
snippets and shell commands presented here were executed on a Debian
GNU/Linux 7, and might have to be adapted to your particular
system. Use them carefully.

## 2 Mock-communities analyses

### 2.1 Synopsis

Swarm's performance was tested on two mock communities each comprising
genome isolates from 49 bacterial and 10 archaeal species: one with
even abundances with 152,523 unique amplicons (1,614,012 raw reads of
average length 254.6 bp), the other with uneven populations with
56,628 unique amplicons (641,541 raw reads of average length 253.9
bp). In both cases, sequencing on the MiSeq platform with 250~bp
paired-end reads was performed following amplification of the V4
region of the 16S rRNA gene with fusion Golay adaptors barcoded on the
reverse end. The forward 16S rRNA primer sequence 515f was used
(GTGNCAGCMGCCGCGGTAA). The reverse primers, barcodes and adaptors were
identical to Caporaso et al. (2011).

Sequences were trimmed for quality and adaptors using Sickle and then
forward and reverse reads were overlapped with PandaSeq (Masella et
al, 2012) insisting on a 50 bp overlap. Strictly identical amplicons
were merged with the command described in Swarm's README, and the two
data sets were subjected to five different stand-alone clustering
methods in their last versions: CD-HIT-454 (Fu et al., 2012), DNAclust
(Ghodsi and Pop, 2011), Swarm (v1.2.3), Usearch (Edgar, 2010) with
presorting of the amplicons by decreasing length (option
-cluster\_fast) and without presorting (options -usersort
-cluster\_smallmem). Different clustering thresholds were used: d = 1
to 20 local differences for Swarm, and t = 1 to 20% global divergence
for the other methods. For each clustering threshold and each
clustering method, the first analysis was done on a fasta file sorted
by decreasing abundance, and repeated 100 times with amplicon input
order randomly shuffled.

The clustering results of the two communities were then evaluated with
three metrics using the known assignments to the 59 species as the
ground truth, which was determined by matching with Usearch against
the known 16S rRNA reference sequences and only using reads that were
within 5% sequence difference using Usearch with the usearch\_global
alignment option. The three metrics used were: *recall*, which
quantifies the extent to which amplicons assigned to the same species
are grouped together in the same OTU (i.e. not over-splitting);
*precision*, which asks if all amplicons in an OTU are assigned to the
same species (i.e. not over-grouping); and the *adjusted Rand index*,
which summarizes both precision and recall as the proportion of pairs
of amplicons that are placed in the same OTU and are from the same
species, but adjusting for the expected proportions through random
chance (Rand, 1971; Hubert and Larabie, 1985). The results were
synthesized in Fig. 2 (uneven community) and Fig. 3 (even community)
using R and the ggplot2 library.

Even = Fusion Golay
Uneven = Mirce

### 2.2 Download the datasets

The cleaned datasets are available and can be downloaded as such:

```
mkdir -p ./data/{even,uneven}
cd ./data/even/
wget http://sbr2.sb-roscoff.fr/download/externe/de/fmahe/even.fasta.bz2
cd ../uneven/
wget http://sbr2.sb-roscoff.fr/download/externe/de/fmahe/uneven.fasta.bz2
```

### 2.3 Dereplication

The two mock-communities were subjected to the same bioinformatics
analyses. For the sake a brevity, only the analysis of the even
community is described here. The MiSeq paired-end reads have already
been quality-filtered, assembled and checked for chimeras. The first
step is to linearize and dereplicate the dataset.

```
cd ./data/even/
RAW_FASTA="19-5ng25CyclesQ5_130624_L001_R1_trim_overlap_nonchim.fasta"
CLEANED_FASTA="even.fasta"

# Linearize and dereplicate
awk 'NR==1 {print ; next} {printf /^>/ ? "\n"$0"\n" : $1} END {print}' "${RAW_FASTA}" | \
grep -v "^>" | grep -v [^ACGTacgt] | sort -d | uniq -c | \
while read abundance sequence ; do
    hash=$(printf "${sequence}" | sha1sum)
    hash=${hash:0:40}
    printf ">%s_%d_%s\n" "${hash}" "${abundance}" "${sequence}"
done | sort -t "_" -k2,2nr -k1.2,1d | sed -e 's/\_/\n/2' > "${CLEANED_FASTA}"
```

Get some statistics:

```
cd ~/Science/Projects/Quince_mock_community/data/even/
paste - - < even.fasta | tr "_" "\t" | \
awk '{l += $2 * length($3) ; sum += $2} END {print NR, sum, l/sum}'
```

143,163 unique amplicons, representing 1,576,869 reads of average
length 254.6 bp.

### 2.4 Taxonomic assignment

Umer Ijaz run the dereplicated dataset and assigned the sequences to
the species level using the pipeline TAXAassign (v0.4) developed in
Christopher Quince's lab, and Blast NCBI's nt database.

```
./TAXAassign.sh -p -c 16 -r 100 -t 60 -m 60 -a "60,70,80,95,95,97" -f even.fasta
```

### 2.5 Clustering Quality Metrics

Shuffle the fasta input-order, produce all clustering results and
compute the five metrics (recall, precision, NMI, Rand index, adjusted
Rand index).

```
# Binaries
SWARM="swarm"
USEARCH="usearch7.0.1001_i86linux32"
CD_HIT="cd-hit-454"
DNACLUST="dnaclust"
PYTHON="python2.7"

# Scripts
FASTA_SHUFFLER="./src/fasta_shuffler.py"
SWARM_BREAKER="./src/swarm_breaker.py"
BUILD_CONFUSION_TABLE="./src/confusion_table.py"
COMPUTE_CLUSTER_METRICS="./src/CValidate.pl"

# Data
FASTA="even.fasta"
TAXONOMIC_ASSIGNMENTS="./results/even/even.spec.ualn"

# Intermediate results
SWARM_RESULTS=$(mktemp)
UCLUST_RESULTS_A=$(mktemp)
UCLUST_RESULTS_A2=$(mktemp)
UCLUST_RESULTS_B=$(mktemp)
UCLUST_RESULTS_B2=$(mktemp)
CD_HIT_RESULTS=$(mktemp)
CD_HIT_RESULTS2=$(mktemp)
DNACLUST_RESULTS=$(mktemp)
CONFUSION_TABLE=$(mktemp)

# Metrics
METRICS="${FASTA/.fasta/.metrics_shuffled}"

# Shuffle
cd ./data/even/
"${PYTHON}" "${FASTA_SHUFFLER}" -i "${FASTA}" -n 100
cp "${FASTA}" "${FASTA/.fasta/_shuffled_000.fasta}"

# Multi-clustering
shuffling=0
echo "method,shuffling,d,recall,precision,nmi,rand,adjustedrand" > "${METRICS}"
# All fasta files
for f in *_shuffled_*.fasta ; do
    # All clustering thresholds
    for i in {1..20} ; do
        identity="0.$(( 100 - $i ))"
        # SWARM
        "${SWARM}" -d ${i} < "${f}" > "${SWARM_RESULTS}" 2> /dev/null
        "${PYTHON}" "${SWARM_BREAKER}" -f "${f}" -s "${SWARM_RESULTS}" -d ${i} 2> /dev/null > "${SWARM_RESULTS2}"
        # UCLUST (no pre-sorting)
        "${USEARCH}" -usersort -cluster_smallmem "${f}" -id "${identity}" -uc "${UCLUST_RESULTS}" > /dev/null 2>&1
        awk 'BEGIN {FS="\t"}
             {
              if ($1 == "S") clusters[$2] = $9
              if ($1 == "H") clusters[$2] = clusters[$2] " " $9
             }
             END {
                 for (cluster in clusters) {
                     print clusters[cluster]
                 }
             }' "${UCLUST_RESULTS_A}" > "${UCLUST_RESULTS_A2}"
        # UCLUST (pre-sorting)
        "${USEARCH}" -cluster_fast "${f}" -id "${identity}" -uc "${UCLUST_RESULTS}" > /dev/null 2>&1
        awk 'BEGIN {FS="\t"}
             {
              if ($1 == "S") clusters[$2] = $9
              if ($1 == "H") clusters[$2] = clusters[$2] " " $9
             }
             END {
                 for (cluster in clusters) {
                     print clusters[cluster]
                 }
             }' "${UCLUST_RESULTS_B}" > "${UCLUST_RESULTS_B2}"
        # CD-HIT-454
        "${CD_HIT}" -i "${f}" -o "${CD_HIT_RESULTS}" -c ${identity} -M 0 > /dev/null 2>&1
        awk 'BEGIN {FS = "[>.]"}
             NR != 1 {printf /^>Cluster/ ? "\n" : $2" "}
             END {printf "\n"}' "${CD_HIT_RESULTS}.clstr" | \
        sed -e 's/ $//' > "${CD_HIT_RESULTS2}"
        # DNACLUST
        "${DNACLUST}" -s "${identity}" -i "${f}" | tr "\t" " " > "${DNACLUST_RESULTS}"
        for CLUSTERING_RESULTS in "${SWARM_RESULTS2}" "${UCLUST_RESULTS_A2}" \
            "${UCLUST_RESULTS_B2}" "${CD_HIT_RESULTS2}" "${DNACLUST_RESULTS}" ; do
            python "${BUILD_CONFUSION_TABLE}" -t "${TAXONOMIC_ASSIGNMENTS}" -s "${CLUSTERING_RESULTS}" > "${CONFUSION_TABLE}"
            METRICS=$(perl "${COMPUTE_CLUSTER_METRICS}" --cfile="${CONFUSION_TABLE}")
            METHOD=${CLUSTERING_RESULTS##*/}
            METHOD=${METHOD%.*}
            echo "${METHOD},${shuffling},${i},${METRICS}" >> "${METRICS}"
        done
    done
    shuffling=$(( $shuffling + 1 ))
done

# Cleaning
rm "${CONFUSION_TABLE}" "${SWARM_RESULTS}" "${SWARM_RESULTS2}" \
    "${UCLUST_RESULTS_A}" "${UCLUST_RESULTS_A2}" \
    "${UCLUST_RESULTS_B}" "${UCLUST_RESULTS_B2}" "${CD_HIT_RESULTS}.clstr" \
    "${CD_HIT_RESULTS2}" "${DNACLUST_RESULTS}" even_shuffled_*.fasta
```

### 2.6 Scripts

Here are the perl and python stand-alone scripts used in the above
shell script.

#### 2.6.1 Shuffling

Change the input-order of the fasta sequences.

```
#!/usr/bin/env python
# -*- coding: utf-8 -*-
"""
    Parse a fasta file and output the entries in a shuffled order.
"""

from __future__ import print_function

__author__ = "Frédéric Mahé <mahe@rhrk.uni-kl.de>"
__date__ = "2012/05/14"
__version__ = "$Revision: 2.0"

import os
import sys
from random import shuffle
from Bio import SeqIO
from Bio.Seq import Seq
from optparse import OptionParser

#**********************************************************************#
#                                                                      #
#                            Functions                                 #
#                                                                      #
#**********************************************************************#

def option_parse():
    """
    Parse arguments from command line.
    """
    desc = """Parse a fasta file and output the entries in a shuffled order."""
    
    parser = OptionParser(
        usage="usage: %prog --input_file FILENAME --number_of_shufflings INT",
        description = desc,
        version = "%prog version 2.0")

    parser.add_option(
        "-i", "--input_file",
        metavar = "<FILENAME>",
        action = "store",
        dest = "input_file",
        help = "set <FILENAME> as input file.")

    parser.add_option(
        "-n", "--number_of_shufflings",
        metavar = "<INT>",
        action = "store",
        dest = "number_of_shuffles",
        type = "int",
        default = 10,
        help = "create <INT> shuffled outputs (default is 10).")

    (options, args) = parser.parse_args()
    
    return options.input_file, options.number_of_shuffles

#**********************************************************************#
#                                                                      #
#                              Body                                    #
#                                                                      #
#**********************************************************************#

if __name__ == '__main__':
    
    # Parse command line options.
    input_file, number_of_shuffles = option_parse()
    basename = os.path.splitext(os.path.abspath(input_file))[0]
    extension = os.path.splitext(input_file)[1]

    # Build a list of fasta entries
    input_format = "fasta"
    with open(input_file, "rU") as input_file:
        records = SeqIO.parse(input_file, input_format)
        records_list = [(record.description, str(record.seq))
                        for record in records]

    # Is this a real fasta file? (this test needs improvement)
    if len(records_list) < 2:
        print("Error: something is wrong with the input file.", file=sys.stderr)
        sys.exit(-1)

    # Create and shuffle a list of indices, and output the fasta
    # entries using that list, repeat n times
    index = range(0, len(records_list))
    zeros = len(str(number_of_shuffles))
    for n in range(0, number_of_shuffles):
        shuffle(index)
        output_file = basename + "_shuffled_" + str(n).zfill(zeros) + extension
        with open(output_file, "w") as output_file:
            for i in index:
                print(">", records_list[i][0], "\n", records_list[i][1], sep="",
                      file=output_file)
        
sys.exit(0)
```

#### 2.6.2 Swarm breaker

Refine the clusters produced by Swarm.

```
#!/usr/bin/env python
# -*- coding: utf-8 -*-
"""
    Detect and break chains of amplicons in a swarm.
"""

from __future__ import print_function

__author__ = "Frédéric Mahé <mahe@rhrk.uni-kl.fr>"
__date__ = "2014/01/30"
__version__ = "$Revision: 1.1"

import os
import sys
import tempfile
import itertools
import subprocess
from operator import itemgetter
from optparse import OptionParser

#*****************************************************************************#
#                                                                             #
#                                  Functions                                  #
#                                                                             #
#*****************************************************************************#


def option_parse():
    """
    Parse arguments from command line.
    """
    desc = """Detect and break chains of amplicons in a swarm. That
    script will search for the swarm binary in /usr/bin/. If swarm is
    installed at a different location, please modify the corresponding
    line in the function run_swarm."""

    parser = OptionParser(usage="usage: %prog -f filename -s filename",
                          description=desc,
                          version="%prog version 1.1")

    parser.add_option("-b", "--binary",
                      metavar="<BINARY>",
                      action="store",
                      default="/usr/bin/swarm",
                      dest="binary",
                      help="swarm binary location. Default is /usr/bin/swarm")

    parser.add_option("-f", "--fasta_file",
                      metavar="<FILENAME>",
                      action="store",
                      dest="fasta_file",
                      help="set <FILENAME> as fasta file.")

    parser.add_option("-s", "--swarm_file",
                      metavar="<FILENAME>",
                      action="store",
                      dest="swarm_file",
                      help="set <FILENAME> as swarm file.")

    parser.add_option("-d", "--differences",
                      metavar="<THRESHOLD>",
                      action="store",
                      type="int",
                      default=1,
                      dest="threshold",
                      help="set local clustering <THRESHOLD>. Default is 1")

    (options, args) = parser.parse_args()
    return options.binary, options.fasta_file, options.swarm_file, options.threshold


def fasta_parse(fasta_file):
    """
    List amplicon ids, abundances and sequences, make a list and a dictionary
    """
    with open(fasta_file, "rU") as fasta_file:
        all_amplicons = dict()
        for line in fasta_file:
            if line.startswith(">"):
                amplicon, abundance = line.strip(">\n").split("_")
            else:
                sequence = line.strip()
                all_amplicons[amplicon] = (int(abundance), sequence)
        return all_amplicons


def swarm_parse(swarm_file):
    """
    List amplicons contained in each swarms, sort by decreasing
    abundance. Sort the list of swarms by decreasing mass and
    decreasing size.
    """
    with open(swarm_file, "rU") as swarm_file:
        swarms = list()
        for line in swarm_file:
            amplicons = [(amplicon.split("_")[0], int(amplicon.split("_")[1]))
                         for amplicon in line.strip().split(" ")]
            # Sort amplicons by decreasing abundance and alphabetical order
            amplicons.sort(key=itemgetter(1, 0), reverse=True)
            top_amplicon, top_abundance = amplicons[0]
            swarm_size = len(amplicons)
            swarm_mass = sum([amplicon[1] for amplicon in amplicons])
            swarms.append([top_amplicon, swarm_mass, swarm_size,
                           top_abundance, amplicons])
        # Sort swarms on mass, size and seed name
        swarms.sort(key=itemgetter(1, 2, 0), reverse=True)
        return swarms


def run_swarm(binary, all_amplicons, swarm, threshold):
    """
    Write temporary fasta files, run swarm and collect the graph data
    """
    swarm_command = [binary, "-b", "-d", str(threshold)]
    with open(os.devnull, "w") as devnull:
        with tempfile.SpooledTemporaryFile() as tmp_fasta_file:
            with tempfile.SpooledTemporaryFile() as tmp_swarm_results:
                for amplicon, abundance in swarm:
                    sequence = all_amplicons[amplicon][1]
                    print(">", amplicon, "_", str(abundance), "\n", sequence,
                          sep="", file=tmp_fasta_file)
                tmp_fasta_file.seek(0)  # rewind to the begining of the file
                proc = subprocess.Popen(swarm_command,
                                        stderr=tmp_swarm_results,
                                        stdout=devnull,
                                        stdin=tmp_fasta_file,
                                        close_fds=True)
                proc.wait()  # usefull or not?
                tmp_swarm_results.seek(0)  # rewind to the begining of the file
                graph_data = [line.strip().split("\t")[1:4]
                              for line in tmp_swarm_results
                              if line.startswith("@")]
                return graph_data


def build_graph(graph_data):
    """
    List pairwise relations in a swarm. Note that not all pairwise
    relations are stored. That's why the graph exploration must always
    start from the most abundant amplicon, and must be reiterated for
    sub-swarms after a breaking.
    """
    graph = dict()
    for line in graph_data:
        ampliconA, ampliconB, differences = line
        if ampliconA in graph:
            graph[ampliconA] += [ampliconB]
        else:
            graph[ampliconA] = [ampliconB]
    return graph


def find_path(graph, start, end, path=[]):
    """
    Recursively explore the graph and find all paths connecting two
    amplicons (http://www.python.org/doc/essays/graphs.html). As the
    graph is not complete, some pairs of amplicon cannot be linked.
    """
    path = path + [start]
    if start == end:
        return path
    if start not in graph:
        return None
    for node in graph[start]:
        if node not in path:
            newpath = find_path(graph, node, end, path)
            if newpath:
                return newpath
    return None


def graph_breaker(amplicons, graph, all_amplicons, ABUNDANT):
    """
    Find deep valleys and cut the graph
    """
    # High peaks to test (starting and ending points)
    top_amplicons = [amplicon[0] for amplicon in amplicons
                     if amplicon[1] >= ABUNDANT]
    # Ending peak is RATIO times higher than the valley
    RATIO = 50
    # Debugging
    print("## OTU ", top_amplicons[0], "\n", "# List potential bridges",
          sep="", file=sys.stderr)
    # Initialize the list of new seeds
    new_swarm_seeds = [top_amplicons[0]]
    # Break if there is no second peak
    if len(top_amplicons) < 2:
        return new_swarm_seeds, graph
    # Loop over the list of top amplicons
    pairs_of_peaks = itertools.combinations(top_amplicons, 2)
    for pair_of_peaks in pairs_of_peaks:
        start_amplicon, end_amplicon = pair_of_peaks
        path = find_path(graph, start_amplicon, end_amplicon)
        # Path can be empty if the relation have been deleted
        if path and len(path) > 1:
            abundances = [int(all_amplicons[node][0]) for node in path]
            # Find the weakest spot
            lowest = min(abundances)
            if lowest != abundances[-1]:
                # LOW VALLEY MODEL (CHANGE HERE)
                if (abundances[-1] / lowest > RATIO / 2 and abundances[0] / abundances[-1] < 10) or abundances[-1] / lowest >= RATIO:
                    # Debugging
                    print(abundances, "\tBREAK!", file=sys.stderr)
                    # Find the rightmost occurence of the lowest point
                    index = len(abundances) - (abundances[::-1].index(lowest) + 1)
                    left_amplicon = path[index-1]
                    right_amplicon = path[index]
                    # Delete the relation from the graph
                    graph[left_amplicon].remove(right_amplicon)
                    # Remove the graph entry if the relation is now empty
                    if not graph[left_amplicon]:
                        del graph[left_amplicon]
                    # Lowest point will be a new swarm seed
                    new_swarm_seeds.append(right_amplicon)
                else:
                    print(abundances, file=sys.stderr)
    return new_swarm_seeds, graph


def swarmer(graph, seed, path=[]):
    """
    Recursively explore the graph and find all amplicons linked to the
    seed
    """
    path = path + [seed]
    if seed in graph:
        for node in graph[seed]:
            path = swarmer(graph, node, path)
    return path


def swarm_breaker(binary, all_amplicons, swarms, threshold):
    """
    Recursively inspect and break the newly produced swarms
    """
    # ARBITRARY PARAMETERS
    ABUNDANT = 100
    # Deal with each swarm
    for swarm in swarms:
        top_amplicon, swarm_mass, swarm_size, top_abundance, amplicons = swarm
        if swarm_size > 2 and top_abundance > ABUNDANT:
            # Run swarm to get the pairwise relationships
            graph_raw_data = run_swarm(binary, all_amplicons, amplicons, threshold)
            # Build the graph of pairwise relationships
            graph = build_graph(graph_raw_data)
            new_swarm_seeds, graph = graph_breaker(amplicons, graph,
                                                   all_amplicons, ABUNDANT)
            # Explore the graph and find all amplicons linked to the seeds
            observed = 0
            new_swarms = list()
            for seed in new_swarm_seeds:
                new_swarm = swarmer(graph, seed)
                observed += len(new_swarm)
                # Give to the new swarms the same structure and
                # re-order them by decreasing abundance
                amplicons = [(amplicon, all_amplicons[amplicon][0])
                             for amplicon in new_swarm]
                amplicons.sort(key=itemgetter(1), reverse=True)
                top_amplicon, top_abundance = amplicons[0]
                swarm_size = len(amplicons)
                swarm_mass = sum([amplicon[1] for amplicon in amplicons])
                new_swarms.append([top_amplicon, swarm_mass,
                                   swarm_size, top_abundance, amplicons])
            # Deal with the new swarms (no need to treat again the
            # first swarm). There will always be at least one swarm in
            # new_swarms.
            print(" ".join(["_".join([amplicon[0], str(amplicon[1])])
                            for amplicon in new_swarms[0][4]]), file=sys.stdout)
            new_swarms.pop(0)
            if new_swarms:
                # Sort the rest of the new swarms by decreasing mass
                # and size. Inject them into swarm_breaker.
                new_swarms.sort(key=itemgetter(1, 2), reverse=True)
                swarm_breaker(binary, all_amplicons, new_swarms, threshold)
        else:
            # Output the swarm
            print(" ".join(["_".join([amplicon[0], str(amplicon[1])])
                            for amplicon in amplicons]), file=sys.stdout)
    return None


def main():
    """
    Hypothesis: chain of amplicons happen among the most abundant
    amplicons of the swarm. The number of chains in a swarm is
    small. The abundances of each sub-swarm centroids are
    comparable. The "valleys" are deep compared to the "peaks". Swarm
    graphs are acyclical, so there is only one path joining two
    amplicons.

    Synopsis: Break bridges as you discover them. Find the weakest
    point in the chain, break on the left of that point and mark it as
    the seed of a new swarm. Repeat the process with the nth most
    abundant amplicon, until all amplicons in the arbitrary range have
    been treated.
    """
    # Parse command line options.
    binary, fasta_file, swarm_file, threshold = option_parse()
    # Load all amplicon ids, abundances and sequences
    all_amplicons = fasta_parse(fasta_file)
    # Load the swarming data
    swarms = swarm_parse(swarm_file)
    # Deal with each swarm
    swarm_breaker(binary, all_amplicons, swarms, threshold)


#*****************************************************************************#
#                                                                             #
#                                     Body                                    #
#                                                                             #
#*****************************************************************************#

if __name__ == '__main__':

    main()

sys.exit(0)
```

#### 2.6.3 Confusion table

Parse the clustering results and the taxonomic assignments results to
build a "confusion table", with each species on one line and one
column per OTU. Several OTUs can be assigned to the same species, but
OTUs should not contain amplicons assigned to different species. Each
cell of the table should contain the total number of reads (from that
OTU and assigned to that species).

```
 #!/usr/bin/env python
# -*- coding: utf-8 -*-

"""
Parse taxonomic assignments and swarm results to produce a confusion
table.
"""

from __future__ import print_function

__author__ = "Frédéric Mahé <mahe@rhrk.uni-kl.fr>"
__date__ = "2013/09/21"
__version__ = "$Revision: 0.1"

# This program is free software: you can redistribute it and/or modify
# it under the terms of the GNU General Public License as published by
# the Free Software Foundation, either version 3 of the License, or
# (at your option) any later version.

# This program is distributed in the hope that it will be useful,
# but WITHOUT ANY WARRANTY; without even the implied warranty of
# MERCHANTABILITY or FITNESS FOR A PARTICULAR PURPOSE.  See the
# GNU General Public License for more details.

# You should have received a copy of the GNU General Public License
# along with this program.  If not, see <http://www.gnu.org/licenses/>.

import sys
from optparse import OptionParser


def option_parser():
    """
    Parse arguments from command line.
    """
    desc = """This program reads a table of taxonomic assignments and
    a swarm clustering file and outputs a confusion table (OTUs vs
    taxonomic assignments)."""

    parser = OptionParser(usage="usage: %prog -t FILENAME -s FILENAME",
                          description=desc,
                          version="%prog version 0.1")

    parser.add_option("-t", "--taxonomic_assignments",
                      metavar="<FILENAME>",
                      action="store",
                      dest="taxonomic_assignments",
                      help="set <FILENAME> as input.")

    parser.add_option("-s", "--swarm_OTUs",
                      metavar="<FILENAME>",
                      action="store",
                      dest="swarm_OTUs",
                      help="set <FILENAME> as input.")

    (options, args) = parser.parse_args()
    return options.taxonomic_assignments, options.swarm_OTUs


def parse_taxonomy(taxonomic_assignments):
    """
    Parse taxonomy assignments
    """
    amplicon2taxonomy = dict()
    taxa = ["Unassigned"]
    with open(taxonomic_assignments, "rU") as taxonomic_assignments:
        for line in taxonomic_assignments:
            amplicon, taxon = line.split()[0:2]
            amplicon, abundance = amplicon.split("_")
            amplicon2taxonomy[amplicon] = (abundance, taxon)
            taxa.append(taxon)
    return amplicon2taxonomy, taxa


def dereplicate_taxa(taxa):
    """
    Dereplicate taxa (list of unique taxon names, and initialized dictionary)
    """
    taxa_list = list(set(taxa))
    taxa_dict = dict(zip(taxa_list, [0] * len(taxa_list)))
    return taxa_list, taxa_dict


def OTU_parser(taxa_dict, taxa_list, amplicon2taxonomy, amplicons):
    """
    Parse each OTU and output one line of the confusion table.
    """
    for amplicon in amplicons:
        try:
            amplicon, abundance = amplicon.split("_")
        except ValueError:
            amplicon, abundance = amplicon, 1
        try:
            abundance, taxon = amplicon2taxonomy[amplicon]
        except KeyError:
            taxon = "Unassigned"
        taxa_dict[taxon] += int(abundance)
    OTU_abundance_per_taxa = [str(taxa_dict[taxon]) for taxon in taxa_list]
    return OTU_abundance_per_taxa


if __name__ == '__main__':

    ## Parse command line arguments
    taxonomic_assignments, swarm_OTUs = option_parser()

    ## Parse taxonomy assignments
    amplicon2taxonomy, taxa = parse_taxonomy(taxonomic_assignments)

    ## Dereplicate taxa
    taxa_list, taxa_dict = dereplicate_taxa(taxa)

    ## Output the table header
    print("OTUs_vs_Taxa", "\t".join(taxa_list), sep="\t", file=sys.stdout)

    ## Parse swarm OTUs
    with open(swarm_OTUs, "rU") as swarm_OTUs:
        for i, line in enumerate(swarm_OTUs):
            amplicons = line.strip().split()
            OTU_abundance_per_taxa = OTU_parser(taxa_dict.copy(), taxa_list,
                                                amplicon2taxonomy, amplicons)
            print(str(i+1), "\t".join(OTU_abundance_per_taxa), sep="\t",
                  file=sys.stdout)

sys.exit(0)
```

#### 2.6.4 Clustering metrics

The perl script CValidate.pl was provided by Christopher Quince and
team collaborators. It computes the different clustering metrics using
the confusion table produced by the above python script.

```
#!/usr/bin/perl

use strict;
use Getopt::Long;

my $tFile;

GetOptions("cfile=s"   => \$tFile,"sfile=s");

my @cluster = ();

open(FILE,"$tFile") or die "Can't open $tFile\n";

my $line = <FILE>;
my $i = 0;
while($line = <FILE>){
  chomp($line);

  my @tokens = split(/\t/,$line);
  shift(@tokens);
  my $j = 0;
 
  foreach my $tok(@tokens){
    #print "$j $tok\n";
    $cluster[$i][$j] = $tok;
    $j++;
  }
  $i++;
}

close(FILE);


printf("%f,%f,%f,%f,%f\n",recall(@cluster),precision(@cluster),nmi(@cluster),randindex(@cluster),adjrandindex(@cluster));

sub precision(){
   my @cluster = @_;
   my $nN = 0;
   my $nC = scalar(@cluster);
   my $nK = scalar(@{$cluster[0]});
   my $precision = 0;

   for(my $i = 0; $i < $nC; $i++){
     my $maxS = 0;

     for(my $j = 0; $j < $nK; $j++){
       if($cluster[$i][$j] > $maxS){
     $maxS = $cluster[$i][$j];
       }
       
       $nN += $cluster[$i][$j];
     }
     $precision += $maxS;
   } 

   return $precision/$nN;
}

sub recall(){
   my @cluster = @_;
   my $nN = 0;
   my $nC = scalar(@cluster);
   my $nK = scalar(@{$cluster[0]});
   my $recall = 0;

   for(my $i = 0; $i < $nK; $i++){
     my $maxS = 0;

     for(my $j = 0; $j < $nC; $j++){
       if($cluster[$j][$i] > $maxS){
     $maxS = $cluster[$j][$i];
       }
       
       $nN += $cluster[$j][$i];
     }
     
     $recall += $maxS;
   } 

   return $recall/$nN;
}

sub choose2{
  my $N = shift;
  my $ret = $N*($N - 1);

  return int($ret/2);
}

sub randindex{
 my @cluster = @_;
 my @ktotals = ();
 my @ctotals = ();
 my $nN = 0;
 my $nC = scalar(@cluster);
 my $nK = scalar(@{$cluster[0]});
 my $cComb = 0;
 my $kComb = 0;
 my $kcComb = 0;
 
 for(my $i = 0; $i < $nK; $i++){
   $ktotals[$i] = 0;
   for(my $j = 0; $j < $nC; $j++){
     $ktotals[$i]+=$cluster[$j][$i];
   }
   $nN += $ktotals[$i];
   $kComb += choose2($ktotals[$i]);
 }
         
  
 for(my $i = 0; $i < $nC; $i++){
   $ctotals[$i] = 0;
   for(my $j = 0; $j < $nK; $j++){
     $ctotals[$i]+=$cluster[$i][$j];
   }
   $cComb += choose2($ctotals[$i]); 
 }

 for(my $i = 0; $i < $nC; $i++){
   for(my $j = 0; $j < $nK; $j++){
     $kcComb += choose2($cluster[$i][$j]);
   }
 }

 my $nComb = choose2($nN);

 return ($nComb - $cComb - $kComb + 2*$kcComb)/$nComb;

}

sub adjrandindex{
 my @cluster = @_;
 my @ktotals = ();
 my @ctotals = ();
 my $nN = 0;
 my $nC = scalar(@cluster);
 my $nK = scalar(@{$cluster[0]});
 my $cComb = 0;
 my $kComb = 0;
 my $kcComb = 0;
 
 for(my $i = 0; $i < $nK; $i++){
   $ktotals[$i] = 0;
   for(my $j = 0; $j < $nC; $j++){
     $ktotals[$i]+=$cluster[$j][$i];
   }
   $nN += $ktotals[$i];
   $kComb += choose2($ktotals[$i]);
 }
         
  
 for(my $i = 0; $i < $nC; $i++){
   $ctotals[$i] = 0;
   for(my $j = 0; $j < $nK; $j++){
     $ctotals[$i]+=$cluster[$i][$j];
   }
   $cComb += choose2($ctotals[$i]); 
 }

 for(my $i = 0; $i < $nC; $i++){
   for(my $j = 0; $j < $nK; $j++){
     $kcComb += choose2($cluster[$i][$j]);
   }
 }

 my $nComb = choose2($nN);
 
 my $temp = ($kComb*$cComb)/$nComb;

 my $ret = $kcComb - $temp;

 return $ret/(0.5*($cComb + $kComb) - $temp);

}


sub nmi{
  my @cluster = @_;
  my @ktotals = ();
  my @ctotals = ();
  my $nN = 0;
  my $nC = scalar(@cluster);
  my $nK = scalar(@{$cluster[0]});
  my $HC = 0.0;
  my $HK = 0.0;

  for(my $i = 0; $i < $nK; $i++){
    $ktotals[$i] = 0;
    for(my $j = 0; $j < $nC; $j++){
      $ktotals[$i]+=$cluster[$j][$i];
    }
    $nN += $ktotals[$i];
  }
         
  
  for(my $i = 0; $i < $nC; $i++){
    $ctotals[$i] = 0;
    for(my $j = 0; $j < $nK; $j++){
      $ctotals[$i]+=$cluster[$i][$j];
    }
    my $dFC = $ctotals[$i]/$nN;
    $HC += -$dFC*log($dFC);
  }

  for(my $i = 0; $i < $nK; $i++){
    my $dFK = $ktotals[$i]/$nN;
    $HK += -$dFK*log($dFK);
  }
  
  
  my $NMI = 0.0;

  for(my $i = 0; $i < $nK; $i++){
    my $NMII = 0.0;

    for(my $j = 0; $j < $nC; $j++){
      my $dF = ($nN*$cluster[$j][$i])/($ctotals[$j]*$ktotals[$i]);
      if($dF > 0.0){
    $NMII += $cluster[$j][$i]*log($dF);
      }
    }
    $NMII /= $nN;
    $NMI += $NMII;
  }

  return (2.0*$NMI)/($HC + $HK);
}
```

### 2.7 Visualization

The results were synthesized in Fig. 2 (uneven community) and Fig. 3
(even community) using R and the ggplot2 library.

Produce a long-form table first:

```
cd ./results/even/
# Produce a long table version
sort -t "," -k1,1d -k2,2n -k3,3n even_clustering.metrics | \
    awk 'BEGIN {
             FS = ","
             OFS = ","
             split("recall precision NMI rand adjustedrand", a, " ")
         }
         {for (i=1; i<=5; i++) print $1, $2, $3, a[i], $(i+3)}' > even_clustering.metrics2
rm even_clustering.metrics
mv even_clustering.metrics2 even_clustering.metrics
```

Then, use this R script to produce the plots:

```
library(ggplot2)
library(reshape)

setwd("./results/even/")
d <- read.table("even_clustering.metrics", dec = ".", sep =",")
colnames(d) <- c("method", "shuffling", "divergence", "metric", "value")

# Divergence values treated as class. Force numerical sorting
d$divergence <- as.character(d$divergence)
d$divergence <- factor(d$divergence,
                       levels=c("1", "2", "3", "4", "5",
                                "6", "7", "8", "9", "10",
                                "11", "12", "13", "14", "15",
                                "16", "17", "18", "19", "20"),
                       ordered=TRUE)

# Subset and rename
d2 <- subset.data.frame(d, d$metric != "NMI" & d$metric != "rand")
levels(d2$method)[4] <- "Swarm"
levels(d2$method)[1] <- "CD-HIT-454"
levels(d2$method)[2] <- "DNAclust"
levels(d2$method)[5] <- "Usearch (no presorting)"
levels(d2$method)[6] <- "Usearch (presorting)"
levels(d2$metric)[1] <- "adjusted Rand index"

# Faceted plot
ggplot(d2, aes(x = divergence, y = value)) +
    geom_boxplot(outlier.size = 1.5) +
    scale_x_discrete(breaks = c("1", "2", "3", "4", "5",
                                "6", "7", "8", "9", "10",
                                "11", "12", "13", "14", "15",
                                "16", "17", "18", "19", "20"),
                              labels=c("1", "", "3", "", "5",
                                       "", "7", "", "9", "", "11",
                                       "", "13", "", "15", "",
                                       "17", "", "19", "")) +
    xlab("divergence (global clustering threshold t in %; or swarm local clustering threshold d)") +
    ylab("metric values") +
    facet_grid(metric ~ method)
ggsave(file = "even_clustering_selected_metrics.pdf", width=16, height=9)

# Compute median values
d4 <- melt(cast(d, metric~divergence~method, median))
colnames(d4) <- c("metric", "divergence", "method", "value")
d4$divergence <- as.character(d4$divergence)
d4$divergence <- factor(d4$divergence,
                        levels = c("1", "2", "3", "4", "5",
                                   "6", "7", "8", "9", "10",
                                   "11", "12", "13", "14", "15",
                                   "16", "17", "18", "19", "20"),
                        ordered=TRUE)
d5 <- subset.data.frame(d4, d4$method != "swarm" & d4$metric != "NMI" & d4$metric != "rand")
levels(d5$method)[4] <- "Swarm"

# Faceted plot using median values
ggplot(d5, aes(x = divergence, y = value)) +
    geom_boxplot() +
    xlab("divergence (% or swarm d value)") +
    ylab("metric values") +
    facet_grid(metric ~ method, scales = "free_y")
ggsave(file = "even_clustering_selected_metrics_medians.pdf", width=16, height=9)

# Output median values to a file
d7 <- cast(d5, metric ~ method ~ divergence)
write.csv(d7, file = "even_median_table.csv", quote = TRUE, eol = "\n", na = "NA")

quit(save = "no")
```

Author: Frédéric Mahé
<mahe@rhrk.uni-kl.de>

Date: [2014-05-12 lun.]

HTML generated by org-mode 6.33x in emacs 23
